# Early Diagnosis of Papillary Carcinoma in a Thyroglossal Duct Cyst: A Multidisciplinary Approach and the Crucial Role of Fine-Needle Aspiration Cytology

**DOI:** 10.7759/cureus.71254

**Published:** 2024-10-11

**Authors:** Margarida Colino, Diana Breda, Bárbara Sepodes, Catarina Melo, Leonor A Barroso

**Affiliations:** 1 Maxillofacial Surgery Department, Unidade Local de Saúde de Coimbra, Coimbra, PRT; 2 Anatomic Pathology Department, Unidade Local de Saúde de Coimbra, Coimbra, PRT; 3 General Surgery Department, Unidade Local de Saúde de Coimbra, Coimbra, PRT

**Keywords:** fine-needle aspiration cytology, multidisciplinary approach, papillary carcinoma, sistrunk procedure, thyroglossal duct cyst, thyroid malignancy

## Abstract

Papillary carcinoma within a thyroglossal duct cyst is a rare and challenging presentation of thyroid malignancy, requiring a nuanced approach to diagnosis and treatment. This report details the case of a 40-year-old male who presented with a two-year history of submental swelling and intermittent respiratory difficulty. Initial imaging suggested a simple thyroglossal duct cyst. However, pathologist-performed fine-needle aspiration cytology (FNAC) revealed atypical cells consistent with papillary carcinoma within the cystic wall. Subsequent imaging uncovered additional suspicious thyroid nodules. A multidisciplinary team recommended total thyroidectomy combined with the Sistrunk procedure to remove the cyst and confirm the nature of the thyroid lesions. Histopathological analysis of the surgical specimens confirmed the presence of primary papillary carcinoma within the thyroglossal duct cyst and benign nodular thyroid hyperplasia. Given the findings, postoperative management involved clinical surveillance rather than adjuvant therapy. This case highlights the critical role of FNAC in the early diagnosis of thyroglossal duct cyst carcinoma and underscores the importance of a multidisciplinary approach in managing such complex cases. The discussion explores the rarity of this condition, the diagnostic challenges it presents, and the therapeutic dilemmas involved, particularly the decision-making process surrounding the extent of surgical intervention. Early diagnosis of papillary carcinoma within a thyroglossal duct cyst is crucial for effective management and patient outcomes. The integration of FNAC, detailed imaging, and multidisciplinary collaboration facilitates accurate diagnosis and appropriate surgical planning. Despite the absence of standardized treatment protocols due to the rarity of this pathology, the case demonstrates that a tailored approach - based on established diagnostic criteria and individualized patient assessment - can lead to successful management. Ongoing research and documentation of similar cases are essential to refine treatment strategies and improve prognostic outcomes for patients with this unusual presentation of thyroid carcinoma.

## Introduction

Thyroglossal duct cysts represent one of the most frequent cervical masses encountered in both adult and pediatric patients, yet occurrences of papillary thyroid carcinoma within these congenital malformations are rare [1]. Embryologically, the thyroid gland migrates through the thyroglossal duct during development, originating from the base of the tongue and traversing the hyoid bone to its final anterior position, anterior to the larynx. Consequently, this pathway is susceptible to ectopic thyroid tissue, leading to cyst formation along its entire trajectory. Papillary thyroid carcinomas within this location can manifest as primary tumors originating from ectopic thyroid tissue or as metastases from primary carcinomas of the thyroid gland [2]. Due to the rarity of this pathology, definitive treatment protocols are lacking, and opinions among authors vary. Consequently, the management of such cases currently relies heavily on the consensus reached by the medical team at each institution.

## Case presentation

**Figure 1:**
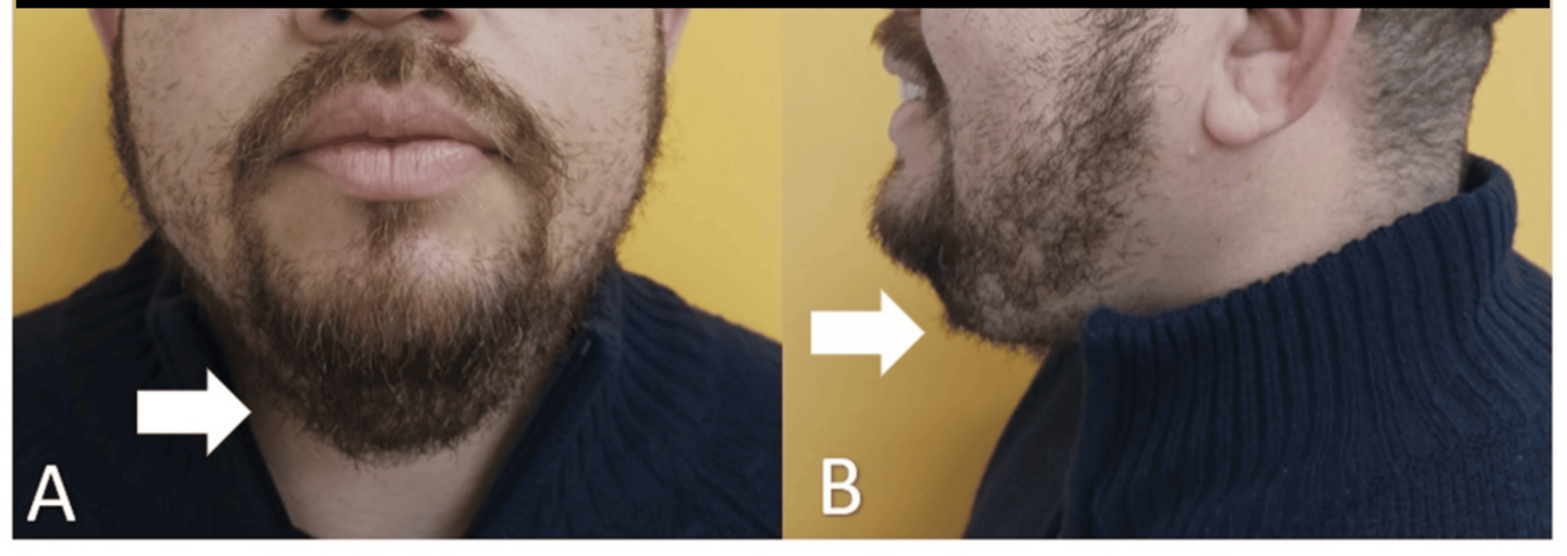
Frontal (A) and lateral (B) preoperative pictures of the patient Preoperative pictures of the patient showing submental swelling (arrows), without any other cervical tumefaction.

A 40-year-old male patient presented with complaints of submental swelling persisting for the past two years, characterized by painless, progressive growth that escalated over the last two months and was accompanied by intermittent respiratory distress (Figures 1A-1B).

**Figure 2:**
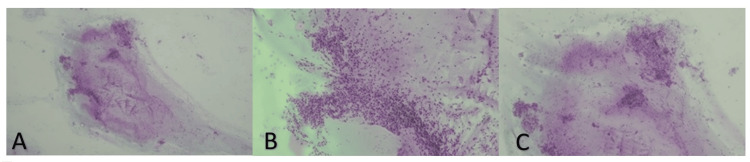
Palpation-guided fine-needle aspiration cytology images Cytologic smears were hypocellular (A), predominantly composed of cells with scant cytoplasm, organized in papillary (B) and follicular arrangements (C). The final diagnosis was made after paraffin embedding and immunohistochemistry, revealing diffuse positivity for TTF-1 and CK19.

A previous cervical ultrasound revealed a lobulated cystic lesion consistent with a thyroglossal duct cyst and a normal thyroid gland without cystic lesions. Physical examination unveiled a well-defined, relatively fixed, soft submental mass extending to the left submandibular region, measuring approximately 7 cm in its longest axis, without palpable cervical adenopathies or thyroid enlargement. Pathologist-performed palpation-guided fine-needle aspiration cytology (FNAC) identified a cystic wall lining epithelium organized into follicles, exhibiting papillae composed of atypical cells. Immunocytochemical staining demonstrated strong and diffuse positivity for CK19 and TTF1. Consequently, the lesion was described as consistent with papillary thyroid carcinoma within a thyroglossal duct cyst (Figures 2A-2C).

**Figure 3:**
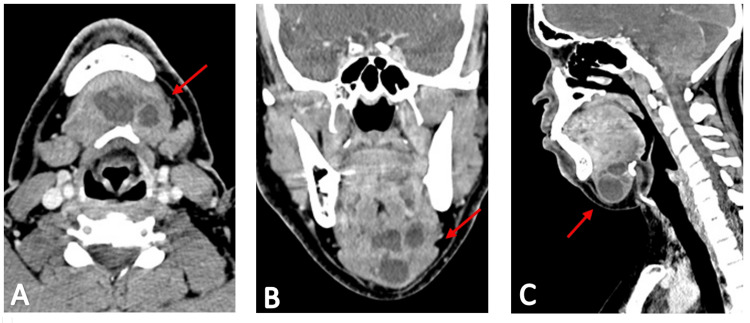
Axial (A), coronal (B), and sagital (C) views of CT scan showing a cystic mass Head and neck CT scan showing a large cystic mass (arrows), compatible with thyroglossal canal cyst. CT: Computed tomography

**Figure 4:**
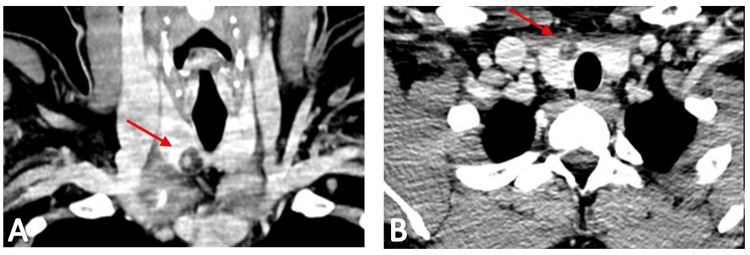
Coronal (A) and axial (B) views of CT scan showing a thyroid gland nodule Head and neck CT scan showing a cystic nodule (arrows) within the right thyroid lobe. CT: Computed tomography

Cervical, thoracic, and abdominopelvic computed tomography (CT) scans were performed to stage the disease accurately. These imaging studies confirmed a lobulated cystic nodular lesion measuring approximately 4.3 cm, located in the submental region (Figures 3A-3C), but also revealed several textural changes in the thyroid gland that were not previously noted (Figures 4A-4B). Subsequent cervical ultrasound identified a heterogeneous nodule within the thyroid gland, predominantly cystic with multiple thick septa and a solid vegetative component measuring 17 x 10 x 13 mm. Another simple cystic formation measuring 3 mm was detected within the gland, exhibiting benign characteristics. FNAC of the thyroid gland nodules was performed under ultrasound guidance for both structures. The cytological examination of the first nodular thyroid lesion revealed cellular smears with a colloid background and isolated and grouped thyroid cells without atypia, along with foamy histiocytes, indicative of a cystic colloid nodule.

**Figure 5:**
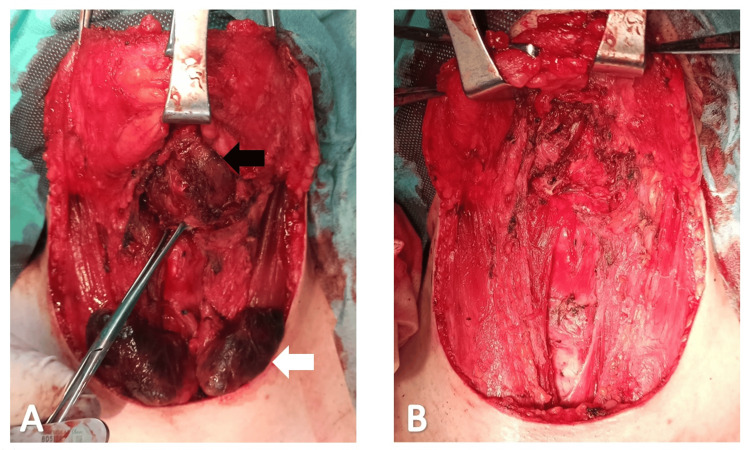
Intraoperative pictures before (A) and after (B) Sistrunk procedure and total thyroidectomy Intraoperative pictures showing both the massive cystic lesion (black arrow) and the thyroid gland (white arrow) before excision (A) and the final immediate postoperative aspect (B).

**Figure 6:**
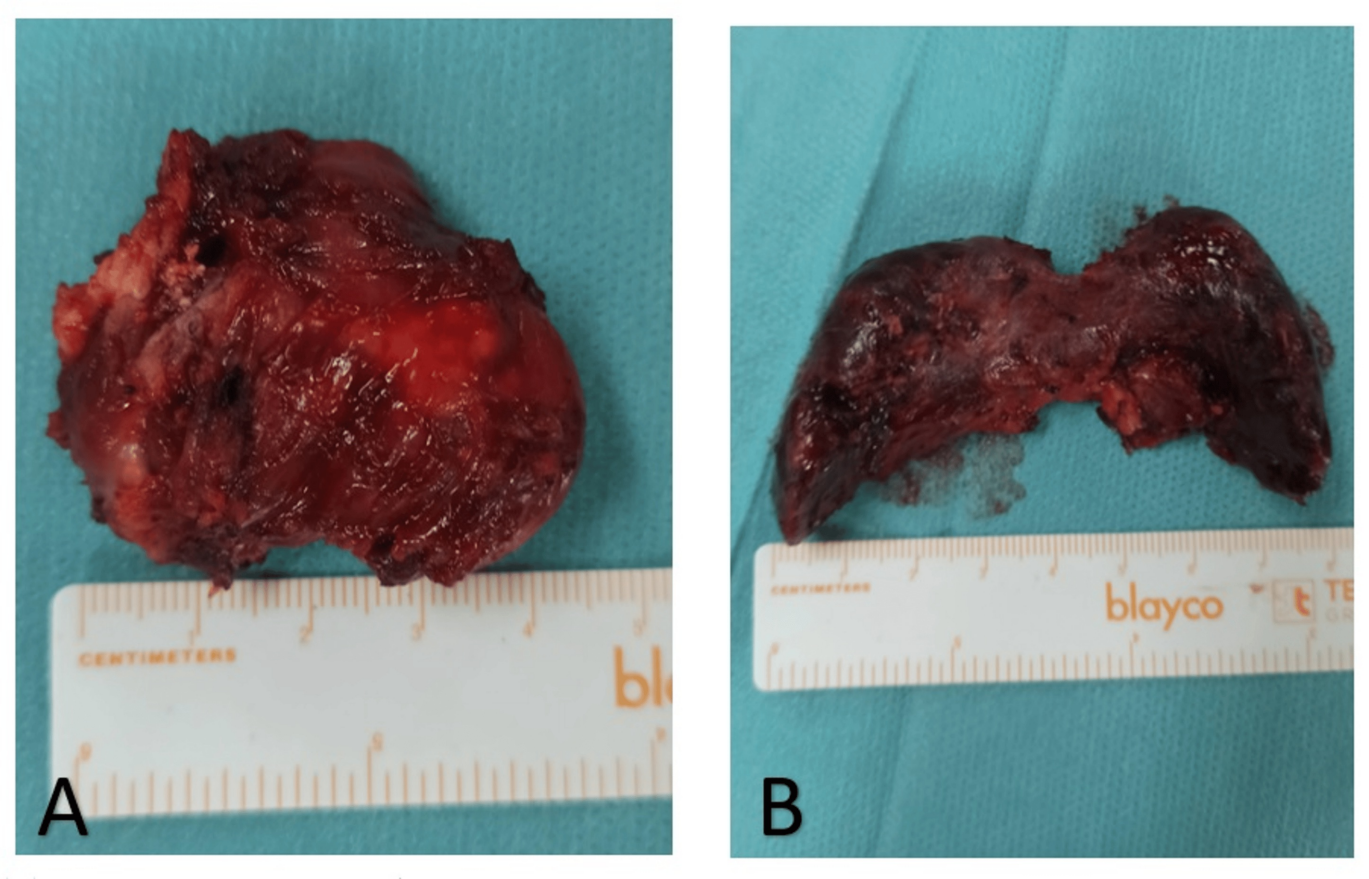
Surgical specimens Pictures of the surgical specimens: (A) cystic lesion and (B) thyroid gland.

Surgical intervention was proposed after a multidisciplinary discussion involving General Surgery, Oncology, and Endocrinology colleagues. Given the presence of a heterogeneous nodule within the thyroid gland alongside papillary carcinoma outside the gland, total thyroidectomy and the Sistrunk procedure were recommended to confirm the nature of the thyroid gland nodule and exclude the possibility of multifocal lesions. The patient underwent the surgical procedure under general anesthesia, with a multidisciplinary team comprising General Surgery and Maxillofacial Surgery surgeons (Figures 5A-5B and Figures 6A-6C). The postoperative course was uneventful.

**Figure 7:**
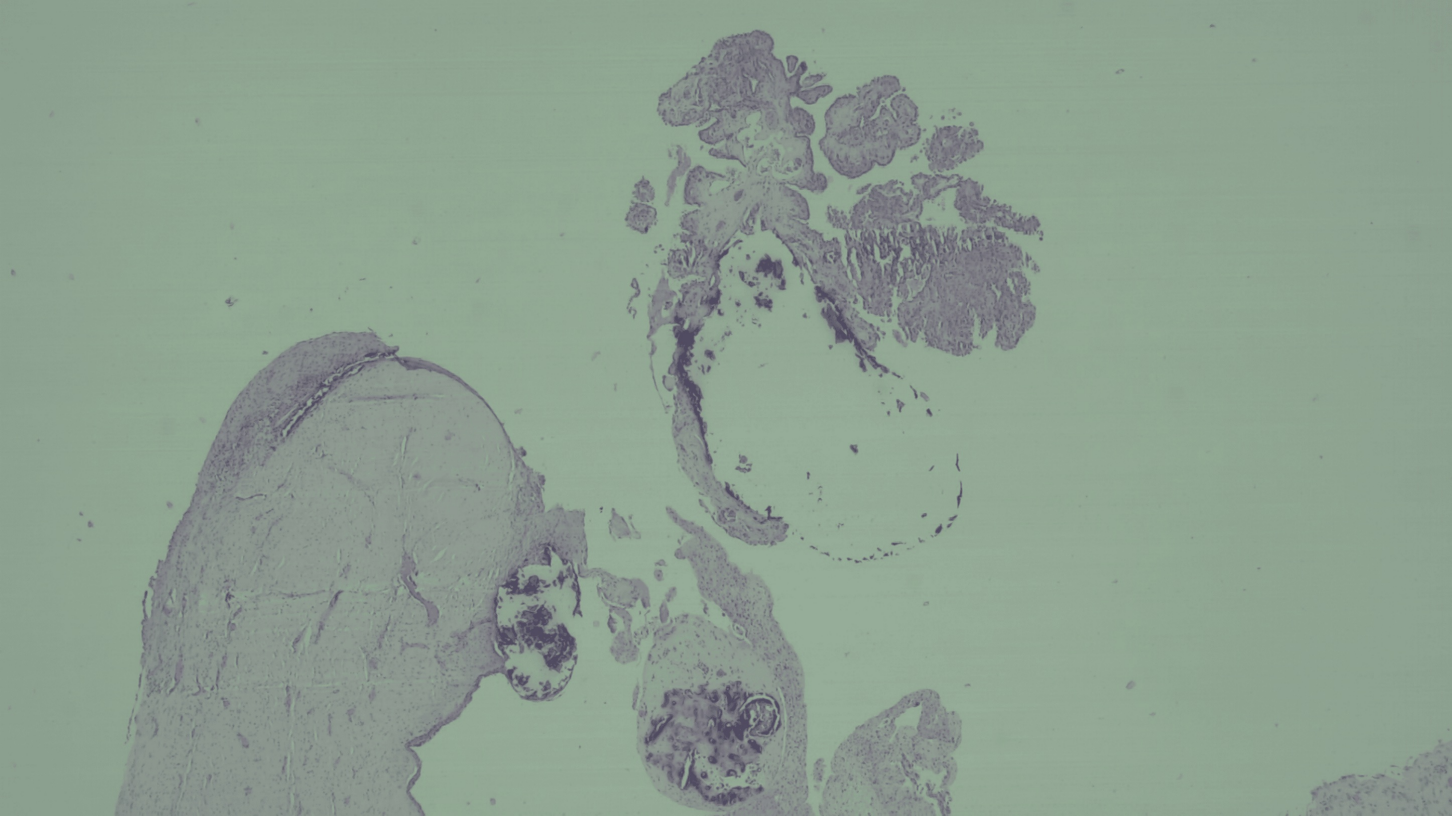
Histopathological examination of the cystic lesion surgical specimen The cystic lesion shows an arborescent papillary proliferation with mainly fibrotic axes covered by sometimes overlapping cells, with elongated nuclei with a ground appearance. The proliferation encompasses multiple calcifications as well as psammoma bodies.

Histopathological examination of the surgical specimens revealed a 16 mm multiloculated cystic formation in the right lobe of the thyroid gland, lacking a specific lining and comprising macrofollicles containing colloid, primarily formed by a single layer of follicular cells with pyknotic, cubic, or flattened nuclei and negative expression of CK19. The smaller thyroid cystic lesion was identified as an 8 mm cavitated lesion, partially containing a non-branching papillary proliferation. A multidisciplinary discussion concluded with a diagnosis of benign nodular thyroid hyperplasia for the thyroid gland. Regarding the surgical specimen resulting from the Sistrunk procedure, histopathological examination revealed a multi-cavitated cystic formation with a non-keratinizing pseudostratified or Malpighian ciliated epithelial lining, accompanied by an inflammatory infiltrate in the wall and an arrangement resembling lymphoid follicles. Additionally, an arborescent papillary proliferation measuring 4 mm, mainly comprising fibrotic axes covered by some overlapping cells with elongated nuclei and a shrunken appearance, showed clear immunoreactivity for CK19 and HBME-1, confirming the diagnosis of primary papillary carcinoma within a thyroglossal duct cyst (Figure 7).

Following surgery, the case was reevaluated in a multidisciplinary meeting involving Oncology, and a decision was made to proceed with clinical surveillance. One year postoperatively, the patient remains well, with no apparent recurrence.

## Discussion

Most papillary carcinomas within thyroglossal duct cysts are diagnosed postoperatively, as the neoplastic process is rarely suspected beforehand. These lesions typically have an indolent presentation, resembling common thyroglossal duct cysts, and patients often exhibit normal thyroid function [3-5]. Consequently, any palpable masses demonstrating fixed, irregular contours, associated adenopathies, or thickening of cyst walls or solid components on imaging studies should raise suspicion of non-benign pathology. Clinical and ultrasound evaluations are essential in suspected thyroglossal duct cyst cases, with FNAC playing a pivotal role in preliminary diagnostic confirmation. While most studies find this technique to have overall sensitivity, specificity, and accuracy above 90%, with high positive and negative predictive values, Rammeh et al. [6] found the lowest specificity (77%) with a false positive rate of 12% for thyroid FNAC. False negatives for this group were papillary carcinomas missed by FNAC, which could be explained partially by the inconstancy of the presence of suggestive nuclear features of papillary carcinomas, such as nuclear grooves and intranuclear cytoplasmic pseudo-inclusions, and also by the frequent presence of a cystic component of these tumors, oftentimes with a thick fibrous capsule, which causes inappropriate interpretation. Tc99 scintigraphy may also aid in detecting ectopic thyroid tissue.

Existing literature suggests two possible origins for these lesions: primary papillary carcinomas developing within the cystic lesion or metastases from primary thyroid tumors. In 1976, Widström et al. [7] established criteria for the diagnosis of primary malignant tumors of thyroglossal duct cysts: neoplastic tissue must be present in the cyst wall; carcinoma must be differentiated from metastases in lymph nodes through histological examination demonstrating squamous or columnar epithelial lining and normal thyroid follicles in the cyst wall; and no malignant lesion should be present in the thyroid or elsewhere.

In most cases, surgical intervention beyond the Sistrunk procedure is deemed unnecessary, mainly when Kristensen's criteria from 1984 are satisfied. These criteria include the presence of normal ectopic thyroid follicles within the cyst, a tumor confined to the cyst wall, and a normal thyroid gland without associated lymphadenopathy. Consequently, the authors advocate for a post-diagnostic thyroid ultrasound and FNAC of any detected nodules. Furthermore, total thyroidectomy, alongside I-131 ablation and thyroid stimulating hormone suppression, is suggested, especially for patients with certain risk factors: male sex, age over 45 years, tumor size larger than 4 cm, prior detection of metastatic lymphadenopathy, history of neck irradiation, evident invasion of the cyst wall, palpable lymph nodes, associated multinodular thyroid gland, or presence of a cold thyroid nodule. In cases where thyroid sparing is preferred, analytical and ultrasound surveillance at 6, 12, and 24 months postoperatively is recommended [3-5].

The case described is uncommon not only due to its rare diagnosis but also because it was diagnosed initially via FNAC. This enabled multidisciplinary discussion and initiated an investigation into the potential origin of the papillary carcinoma. Although it initially appeared to be primary to the cyst, secondary origins needed to be ruled out. Despite the benign FNAC results for the thyroid nodules, heterogeneous imaging findings left the team uneasy. Consequently, total thyroidectomy was decided upon from the outset, concurrent with the Sistrunk procedure. Following histopathological findings indicating carcinoma confined to the cystic wall and benign thyroid gland nodules, further I-131 treatment was deferred, with clinical surveillance maintained.

## Conclusions

Diagnosing papillary carcinoma within a thyroglossal duct cyst presents a rare and intriguing clinical scenario. Despite its uncommon nature, this case underscores the importance of considering neoplastic processes in the differential diagnosis of cervical masses, particularly those associated with atypical features in imaging studies. Multidisciplinary collaboration among surgical, oncological, and pathology teams is essential for accurate diagnosis and appropriate management decisions. While treatment protocols remain undefined due to the rarity of this pathology, surgical excision, guided by established criteria, offers a promising approach. Furthermore, the successful management of such cases highlights the significance of ongoing clinical surveillance and interdisciplinary communication to ensure optimal patient outcomes. Continued research and reporting of similar cases will further enhance our understanding and management of this unique presentation of papillary carcinoma.
